# 550. Effectiveness of Remdesivir for Treatment of COVID-19 in the Outpatient Setting

**DOI:** 10.1093/ofid/ofad500.619

**Published:** 2023-11-27

**Authors:** Vinny P Vo, Shu Lu, Madiha Shah, Hien Nguyen

**Affiliations:** VA Northern California Health Care System, Sacramento, California; VA Northern California Health Care, Folsom, California; VA Northern California Health Care System, Sacramento, California; VA Northern California Health Care System / UC Davis Health, Mather, California

## Abstract

**Background:**

Remdesivir (REM) was the first FDA-approved treatment for COVID-19. A clinical trial evaluated the effectiveness of REM for the outpatient treatment of COVID-19, showing an 87% lower risk of hospitalization and death when given within the first 7 days of infectious symptoms in confirmed infection. "Real-world" studies evaluating the outpatient use of REM when Omicron was the predominant variant have not been published. We aim to evaluate the use of REM in the outpatient setting (REM-OP) at our institution.

**Methods:**

VA Northern California Health Care System developed an outpatient clinic for the treatment of COVID-19 after FDA approval of REM-OP in January 2022. Patients had to meet institutional criteria for outpatient management of COVID-19 with REM, including a confirmed positive PCR test, lack of supplemental oxygen, risk for severe disease, and reliable transportation. We retrospectively evaluated patients given REM-OP from 1/21/2022 - 413/2022 when Omicron was the predominant variant in our region. Data were obtained using a data query of outpatient REM prescriptions. Subsequently, the administrations of REM were verified and patient variables, including risk factors for progression to severe disease, COVID vaccination, history of previous infection, and subsequent hospitalization or death within 30 days, were collected via manual chart review.

**Results:**

88 patients received REM-OP with a mean age of 70.0 (SD +/- 11.4) with a predominantly male (92%) and white (67%) population. Most patients had received a least one dose of COVID-19 vaccination but 17% were unvaccinated. The most common risk factors for progression to severe disease included age (75% were over age 65), diabetes (45%), hypertension (64.8%), and cardiovascular disease (31.8%). 98% of patients met at least one risk factor for progression to severe disease and 95% completed REM-OP. 93% of patients received REM within 7 days of symptom onset. Of the 88 patients who were treated, 5 patients were admitted within 30 days (2/5 were due to COVID-19 infection), and 2 patients died (1 of which was attributed to COVID-19).

Patient Characteristics

**Figure:**
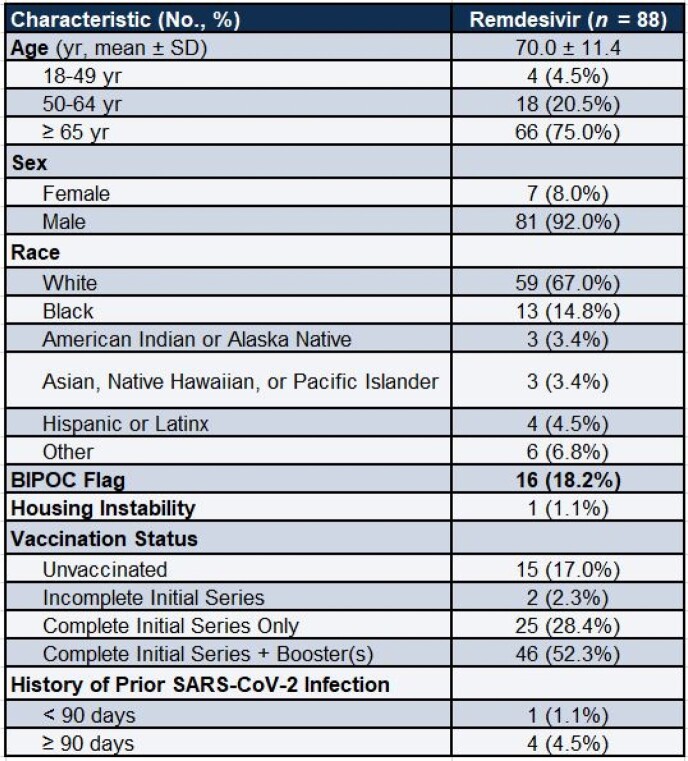

Comparison to PINETREE Clinical Trial

**Figure:**
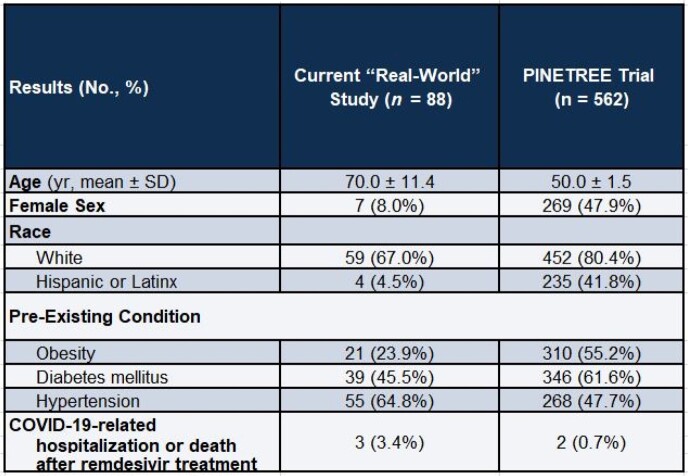

**Conclusion:**

REM-OP during the Omicron surge in our single-center retrospective study had comparable effectiveness to its use in a clinical trial during the Delta surge.

**Disclosures:**

**All Authors**: No reported disclosures

